# Implementation Gaps in US Syringe Services Programs, 2022

**DOI:** 10.1001/jamanetworkopen.2025.22764

**Published:** 2025-07-23

**Authors:** Jamie L. Humphrey, Claire N. Strack, Sheila V. Patel, McKinley E. Saunders, Lynn D. Wenger, Ricky N. Bluthenthal, Paul LaKosky, Stephanie Prohaska, Alex H. Kral, Barrot H. Lambdin

**Affiliations:** 1 RTI International, Research Triangle Park, North Carolina; 2 Keck School of Medicine, University of Southern California, Los Angeles; 3 North American Syringe Exchange Network, Tacoma, Washington

## Abstract

This cross-sectional study examines implementation gaps in syringe service programs by US Census division, urbanicity, and level of need.

## Introduction

Syringe services programs (SSPs) are an effective, cost-saving approach for delivering a variety of services (eg, drug use supplies, overdose education, and naloxone distribution) to reduce hepatitis C virus (HCV), HIV, and opioid overdose fatalities among people who use drugs.^1^ Current information regarding SSP implementation levels in the US and whether SSPs are located in counties with the highest need is insufficient. Using a comprehensive dataset of SSPs in the US, this study characterizes SSP implementation gaps across Census divisions, urbanicity, and levels of need.

## Methods

This research was approved as non–human participants research by the institutional review board of RTI International and follows the STROBE reporting guidelines for cross-sectional studies. We performed a cross-sectional analysis of all 626 SSPs known to be operating in the US in 2022 using the proprietary dataset, Syringe Services Programs in the US (SSPUS), 2022 (eMethods in Supplement 1).^2^ We geocoded 613 (97.9%) SSP headquarter locations to county boundaries and Census divisions. County-level urbanicity was measured using a 3-tier variable.^3^ To account for the underlying need for an SSP within a county, we appended county-level rates per 100 000 residents of 2020 HCV mortality,^4^ 2021 HIV incidence,^5^ and 2021 drug overdose mortality (eTable in Supplement 1).^4^ These measures were lagged to ensure the level of need predated SSPUS data from 2022, and categorized into tertiles so that counties were designated as having a low, moderate, or high rate.

We calculated the percentage of counties that contained at least 1 SSP across different measures of need within each Census division and level of urbanicity. χ^2^ analyses were used to compare the differences in proportions of counties with and without SSPs across each measure of need. A 2-sided *P* < .05 was used to determine statistical significance. Data were analyzed with R statistical software version 4.3.1 (R Project for Statistical Computing).

## Results

In 2022, 613 SSPs were headquartered in 404 of 3221 US counties (12.5%). Nationally, 856 of 1047 high HCV need counties (81.8%), 465 of 669 high HIV need counties (69.5%), and 790 of 1044 high overdose need counties (75.7%) did not have an SSP (Table 1). Overall, counties with high HCV mortality were more likely than counties with low HCV mortality to have SSPs (191 counties [18.2%] vs 95 counties [9.1%]). Similar patterns emerged for HIV incidence (204 high-incidence counties [30.5%] vs 28 low-incidence counties [4.2%]) and drug overdose mortality (254 high-mortality counties [24.3%] vs 34 low-mortality counties [3.3%]). SSP implementation in high-need vs low-need counties were generally consistent across divisions.

**Table 1.** Prevalence of Counties With and Without 1 or More SSP Disaggregated by Need Within Census Divisions, 2022

Census division	Counties, No. (%)								
	HCV mortality rate^a^			HIV incidence rate^a^			Drug overdose mortality rate^a^		
	Low	Medium	High	Low	Medium	High	Low	Medium	High
New England									
SSP	20 (48.8)	9 (42.9)	2 (40.0)	2 (22.2)	7 (41.2)	11 (91.7)	0	6 (35.3)	25 (50.0)
No SSP	21 (51.2)	12 (57.1)	3 (60.0)	7 (77.8)	10 (58.8)	1 (8.3)	0	11 (64.7)	25 (50.0)
* P* value^b^	.87			.001			.58		
Middle Atlantic									
SSP	14 (12.4)	5 (16.1)	2 (33.3)	0	1 (2.5)	19 (35.2)	1 (4.8)	9 (15.0)	11 (15.9)
No SSP	99 (87.6)	26 (83.9)	4 (66.7)	0	39 (97.5)	35 (64.8)	20 (95.2)	51 (85.0)	58 (84.1)
* P* value^b^	.33			<.001			.42		
East North Central									
SSP	32 (11.0)	26 (22.8)	10 (32.3)	6 (5.0)	8 (10.3)	34 (50.8)	5 (6.1)	22 (11.8)	41 (24.4)
No SSP	260 (89.0)	88 (77.2)	21 (67.7)	115 (95.0)	70 (89.7)	33 (49.3)	77 (93.9)	165 (88.2)	127 (75.6)
* P* value^b^	<.001			<.001			<.001		
West North Central									
SSP	4 (1.5)	8 (2.9)	4 (5.1)	2 (0.7)	3 (2.1)	7 (20.0)	10 (2.2)	4 (3.5)	2 (4.8)
No SSP	261 (98.5)	267 (97.1)	74 (94.9)	280 (99.3)	139 (97.9)	28 (80.0)	451 (97.8)	111 (96.5)	40 (95.2)
* P* value^b^	.19			<.001			.48		
South Atlantic									
SSP	21 (10.0)	28 (11.1)	27 (21.4)	0	9 (8.3)	58 (24.3)	6 (5.7)	18 (8.3)	52 (19.5)
No SSP	189 (90.0)	224 (88.9)	99 (78.6)	31 (100.0)	99 (91.7)	181 (75.7)	99 (94.3)	198 (91.7)	215 (80.5)
* P* value^b^	.005			<.001			<.001		
East South Central									
SSP	0	9 (9.2)	33 (16.0)	11 (15.7)	3 (8.6)	10 (13.3)	0	4 (4.5)	38 (20.0)
No SSP	60 (100.0)	89 (90.8)	173 (84.0)	59 (84.3)	32 (91.4)	65 (86.7)	85 (100.0)	85 (95.5)	152 (80.0)
* P* value^b^	.002			.60			<.001		
West South Central									
SSP	1 (3.7)	2 (1.8)	13 (3.9)	0	0	15 (12.9)	3 (1.5)	7 (4.1)	6 (6.1)
No SSP	26 (96.3)	111 (98.2)	317 (96.1)	30 (100.0)	140 (100.0)	101 (87.1)	197 (98.5)	165 (95.9)	92 (93.9)
* P* value^b^	.55			<.001			.10		
Mountain									
SSP	0	17 (15.9)	42 (29.2)	4 (3.4)	14 (20.6)	22 (78.6)	1 (1.6)	15 (13.5)	42 (41.2)
No SSP	30 (100.0)	90 (84.1)	102 (70.8)	113 (96.6)	54 (79.4)	6 (21.4)	63 (98.4)	96 (86.5)	60 (58.8)
* P* value^b^	<.001			<.001			<.001		
Pacific									
SSP	3 (33.3)	11 (30.6)	58 (47.9)	3 (33.3)	13 (31.7)	28 (65.1)	8 (30.8)	27 (35.1)	37 (63.8)
No SSP	6 (66.7)	25 (69.4)	63 (52.1)	6 (66.7)	28 (68.3)	15 (34.9)	18 (69.2)	50 (64.9)	21 (36.2)
* P* value^b^	.15			.006			.001		
National									
SSP	95 (9.1)	115 (11.0)	191 (18.2)	28 (4.2)	58 (8.7)	204 (30.5)	34 (3.3)	112 (10.7)	254 (24.3)
No SSP	952 (90.9)	932 (89.0)	856 (81.8)	641 (95.8)	611 (91.3)	465 (69.5)	1010 (96.7)	932 (89.3)	790 (75.7)
* P* value^b^	<.001			<.001			<.001		

Abbreviations: HCV, hepatitis C virus; SSP, syringe services program.

^a^ The cutoff points defining low, medium, and high for HCV mortality rates, HIV incidence rates, and drug overdose mortality rates are shown in the eTable in Supplement 1.

^b^ See the Methods section for definitions of statistical significance.

Most urban counties (61 of 68 counties [89.7%]) contained 1 or more SSPs, whereas SSPs were present in only 208 of 1098 suburban counties (18.9%) and 132 of 1975 rural counties (6.7%). In nonurban areas, counties with low HCV and drug overdose mortality rates were more likely than counties with high mortality rates to not have an SSP (Table 2).

**Table 2.** Prevalence of Counties With and Without 1 or More SSP Disaggregated by Need Within Levels of Urbanicity, 2022

Urbanicity	Counties, No. (%)								
	HCV mortality rate^a^			HIV incidence rate^a^			Drug overdose mortality rate^a^		
	Low	Medium	High	Low	Medium	High	Low	Medium	High
Urban (n = 68)									
SSP	13 (81.3)	20 (90.9)	28 (93.3)	0	0	61 (91.0)	6 (66.7)	19 (86.4)	36 (97.3)
No SSP	3 (18.8)	2 (9.1)	2 (6.7)	0	1 (100.0)	6 (9.0)	3 (33.3)	3 (13.6)	1 (2.7)
* P* value^b^	.43			.003			.02		
Suburban (n = 1098)									
SSP	60 (13.8)	63 (19.2)	85 (25.3)	3 (3.2)	31 (18.3)	131 (28.9)	18 (7.7)	67 (16.6)	122 (26.6)
No SSP	374 (86.2)	265 (80.8)	251 (74.7)	91 (96.8)	138 (81.7)	322 (71.1)	215 (92.3)	336 (83.4)	336 (73.4)
* P* value^b^	<.001			<.001			<.001		
Rural (n = 1975)									
SSP	22 (3.7)	32 (4.6)	78 (11.5)	25 (4.4)	27 (5.6)	10 (7.9)	10 (1.3)	26 (4.2)	96 (17.5)
No SSP	575 (96.3)	665 (95.4)	603 (88.5)	550 (95.7)	456 (94.4)	117 (92.1)	792 (98.7)	593 (95.8)	453 (82.5)
* P* value^b^	<.001			.24			<.001		

Abbreviations: HCV, hepatitis C virus; SSP, syringe services program.

^a^ The cutoff points defining low, medium, and high for HCV mortality rates, HIV incidence rates, and drug overdose mortality rates are shown in the eTable in Supplement 1.

^b^ See the Methods section for definitions of statistical significance.

## Discussion

In this cross-sectional study of the first comprehensive and spatially referenced dataset of SSPs in the US,^2^ we found substantial SSP implementation gaps throughout the US and significant variation in SSP implementation by level of need in a county. Although counties with a higher need are more likely to have an SSP, the overall levels of implementation remain low, with 69.5% to 81.8% of high-need counties lacking an SSP. Addressing implementation gaps in high-need areas remains critically important to improving health in those communities. Our finding that SSP availability declined with decreasing urbanicity in a dose-related manner aligns with other studies examining the distribution of US SSPs.^6^ Considering unprecedented increases in HCV and HIV infections and overdose mortality in rural communities, more SSPs are needed in nonurban areas to address these critical needs. Study limitations include that the SSPUS may not represent all SSPs as some might want to remain anonymous, and the data represent the county in which SSPs were headquartered, not all counties they served.

## Notes

Supplement 1.
eMethods.
**eTable.** Cut-points used to characterize counties as having a low, moderate, or high rate for each county-level measure of need
eReferences

Supplement 2.
Data Sharing Statement
