# Error in Results

**DOI:** 10.1001/jamanetworkopen.2025.33066

**Published:** 2025-08-19

In the Research Letter titled “Implementation Gaps in US Syringe Services Programs, 2022,”^1^ published July 23, 2025, there was an error in the description of the association of low hepatitis C virus (HCV) and drug overdose mortality rates with the presence of a syringe services program (SSP). This should have read, “In nonurban areas, counties with low HCV and drug overdose mortality rates were more likely than counties with high mortality rates to not have an SSP.” This article has been corrected.^1^
