# Supplementary material for: Implementation Gaps in US Syringe Services Programs, 2022
Source: JAMA Netw Open. 2025 Jul 23;8(7):e2522764. doi: 10.1001/jamanetworkopen.2025.22764 (PMC12287829; doi:10.1001/jamanetworkopen.2025.22764)
Supplement: Supplement 1. — eMethods. eTable. Cut-points used to characterize counties as having a low, moderate, or high rate for each county-level measure of need eReferences [file jamanetwopen-e2522764-s001.pdf]

Supplemental Online Content

Humphrey JL, Strack CN, Patel SV, et al. Implementation gaps in US syringe services programs, 2022. *JAMA Netw Open*. 2025;8(7):e2522764.
doi:10.1001/jamanetworkopen.2025.22764

eMethods.

eTable. Cut-points used to characterize counties as having a low, moderate, or high rate for each county-level measure of need

eReferences

This supplemental material has been provided by the authors to give readers additional information about their work.

© 2025 Humphrey JL et al. *JAMA Network Open*.

eMethods

Data

Syringe Services Programs in the U.S.

The Syringe Services Programs in the U.S., 2022 (SSPUS) is a proprietary dataset of all unique SSPs known to be operating in the U.S. in 2022 (n=626). Since 2019,[1-4] the SSPUS has been constructed annually from several public and private sources: (1) SSPs that were publicly listed in the North American Syringe Exchange Network (NASEN) directory, (2) SSPs that are not publicly listed in NASEN's directory but agreed to be contacted for research purposes, (3) SSPs belonging to NASEN's buyers' club that are not in the directory, (4) SSPs that are not in NASEN's directory and do not participate in the Buyer's Club, but are known to NASEN, (5) SSPs that responded to any of the prior annual National Survey of Syringe Services Programs (NSSSP) conducted by RTI International[5-8] and are not in NASEN's directory, and (6) other SSPs proactively identified through SSP networks by the RTI International project team and partners.

The SSPUS may not represent all SSPs as some programs might prefer to remain anonymous. However, since 2019, we have proactively built the SSPUS using multiple approaches, and the identification of existing, but previously unknown, programs has slowed in recent years.

County-Level Needs Variables

Hepatitis C virus (HCV) mortality and drug overdose mortality rates per 100,000 county population were obtained from HepVu (https://hepvu.org/resources/#/);[9] HIV incidence rates per 100,000 county population was obtained from AIDSvu (https://aidsvu.org/resources/#/datasets).[10] We used tertiles to categorize counties as having a low, moderate, or high rate. The cut-points for each categorization are provided in eTable 1. By accessing these publicly available data and using the cut-points below, policymakers and public health departments can identify counties with higher need for SSP services.

eTable. Cut-points used to characterize counties as having a low, moderate, or high rate for each county-level measure of need.

County-Level Measures of Need	Cut-Points
**HCV Mortality Rate per 100k, 2020**	
Low	0.5 - 2.6
Moderate	2.7 - 4.0
High	4.1 - 50.5
**HIV Incidence Rate per 100k, 2021**	
Low	0.0 - 0.0
Moderate	0.1 - 6.0
High	6.1 - 104.0
**Drug Overdose Mortality Rate per 100k, 2021**	
Low	5.0 - 19.8
Moderate	19.9 - 31.6
High	31.7 - 164.8

County-Level Urbanicity

We constructed a three-tier, county-level measure of urbanicity from the NCHS Urban-Rural Classification Scheme,[11] , following guidance from the Pew Research Center.[12] Urban counties are

© 2025 Humphrey JL et al. *JAMA Network Open*.

located in the 53 metropolitan areas with at least a million people; about 31 % of people residing in the US live in these counties. Suburban and small metro counties include those outside the core metro areas, referred to as “large fringe metro,” “medium metro” and “small metro” counties in the NCHS classification system. About half of US residents (55%) live in suburban counties. Rural counties are located in non-metropolitan areas. With a median population size of 16,535, only 14% of US residents live in them. By accessing these publicly available data and using the methods described above, policymakers and public health departments can identify urban, suburban, and rural counties.

Analysis

Linking SSPUS Data to Counties

We created a county-level dataset by geocoding 613 (97.9%) SSP headquarter locations to Census Bureau cartographic county boundaries. Addresses were not available for 13 SSPs and were not geocoded. We cross-walked counties to Census Bureau divisions (n=9); SSPs located in Puerto Rico were assigned to the South Atlantic division based on proximity.

© 2025 Humphrey JL et al. *JAMA Network Open*.

eReferences

1. Lambdin BH, Humphrey JL, Wenger LD, Saunders ME, Strack CN, Bluthental RN, et al. Syringe Services Programs in the U.S., 2021. RTI International; 2022.
2. Lambdin BH, Humphrey JL, Wenger LD, Saunders ME, Strack CN, Bluthental RN, et al. Syringe Services Programs in the U.S., 2019-2020. RTI International; 2021.
3. Patel SV, Humphrey JL, Prohaska S, Rodriguez Borja I, Saunders ME, Strack CN, et al. Syringe Services Programs in the U.S., 2022. RTI International; 2024.
4. Patel SV, Humphrey JL, Prohaska S, Rodriguez Borja I, Saunders ME, Strack CN, et al. Syringe Services Programs in the U.S., 2023. RTI International; 2024.
5. Lambdin BH, Humphrey JL, Wenger LD, Saunders ME, Strack CN, Bluthenthal R, et al. Program and Operational Characteristics of Syringe Services Programs—Data from the National Survey of Syringe Services Programs, 2019 and 2020. RTI International; 2024.
6. Lambdin BH, Humphrey JL, Wenger LD, Saunders ME, Strack CN, Bluthenthal R, et al. Program and Operational Characteristics of Syringe Services Programs—Data from the National Survey of Syringe Services Programs, 2021. RTI International; 2024.
7. Lambdin BH, Patel SV, Humphrey JL, Wenger LD, Saunders ME, Strack CN, et al. Program and Operational Characteristics of Syringe Services Programs—Data from the National Survey of Syringe Services Programs, 2022. RTI International; 2023.
8. Lambdin BH, Patel SV, Humphrey JL, Wenger LD, Saunders ME, Strack CN, et al. Program and Operational Characteristics of Syringe Services Programs—Data from the National Survey of Syringe Services Programs, 2023. RTI International; 2024.
9. Emory University. HepVu (hepvu.org). In: Health RSoP, editor.
10. Sullivan P, Woodyatt C, Koski C, Pembleton E, McGuinness P, Taussig J, et al. A data visualization and dissemination resource to support HIV prevention and care at the local level: analysis and uses of the AIDSVu Public Data Resource. Journal of medical Internet research. 2020;22(e23173).
11. Ingram DD, SJ. F. 2013 NCHS Urban-Rural Classification Scheme for Counties. Vital Health Statistics. 2014;2(166).
12. Parker K, Horowitz JM, Brown A, Fry R, Cohn D, Igielnik R. What Unites and Divides Urban, Suburban and Rural Communities: Pew Research Center,; May 2018 Contract No.: Document Number|.

© 2025 Humphrey JL et al. *JAMA Network Open*.
