# Supplementary material for: Implementation Gaps in US Syringe Services Programs, 2022
Source: JAMA Netw Open. 2025 Jul 23;8(7):e2522764. doi: 10.1001/jamanetworkopen.2025.22764 (PMC12287829; doi:10.1001/jamanetworkopen.2025.22764)
Supplement: Supplement 2. — Data Sharing Statement [file jamanetwopen-e2522764-s002.pdf]

Data Sharing Statement

Humphrey. Implementation Gaps in US Syringe Services Programs, 2022. *JAMA Netw Open*. Published July 23, 2025. doi:10.1001/jamanetworkopen.2025.22764

Data
Data available: No
